# Tiny Organs, Big Impact: How Microfluidic Organ-on-Chip Technology Is Revolutionizing Mucosal Tissues and Vasculature

**DOI:** 10.3390/bioengineering11050476

**Published:** 2024-05-10

**Authors:** Ishita Dasgupta, Durga Prasad Rangineni, Hasan Abdelsaid, Yixiao Ma, Abhinav Bhushan

**Affiliations:** Department of Biomedical Engineering, Illinois Institute of Technology, Chicago, IL 60616, USA; idasgupta@hawk.iit.edu (I.D.); drangineni@hawk.iit.edu (D.P.R.); habdelsaid@hawk.iit.edu (H.A.); yma60@iit.edu (Y.M.)

**Keywords:** organ-on-chip, epi mucosa, disease models, tissue engineering, cellular engineering, microfluidics

## Abstract

Organ-on-chip (OOC) technology has gained importance for biomedical studies and drug development. This technology involves microfluidic devices that mimic the structure and function of specific human organs or tissues. OOCs are a promising alternative to traditional cell-based models and animals, as they provide a more representative experimental model of human physiology. By creating a microenvironment that closely resembles in vivo conditions, OOC platforms enable the study of intricate interactions between different cells as well as a better understanding of the underlying mechanisms pertaining to diseases. OOCs can be integrated with other technologies, such as sensors and imaging systems to monitor real-time responses and gather extensive data on tissue behavior. Despite these advances, OOCs for many organs are in their initial stages of development, with several challenges yet to be overcome. These include improving the complexity and maturity of these cellular models, enhancing their reproducibility, standardization, and scaling them up for high-throughput uses. Nonetheless, OOCs hold great promise in advancing biomedical research, drug discovery, and personalized medicine, benefiting human health and well-being. Here, we review several recent OOCs that attempt to overcome some of these challenges. These OOCs with unique applications can be engineered to model organ systems such as the stomach, cornea, blood vessels, and mouth, allowing for analyses and investigations under more realistic conditions. With this, these models can lead to the discovery of potential therapeutic interventions. In this review, we express the significance of the relationship between mucosal tissues and vasculature in organ-on-chip (OOC) systems. This interconnection mirrors the intricate physiological interactions observed in the human body, making it crucial for achieving accurate and meaningful representations of biological processes within OOC models. Vasculature delivers essential nutrients and oxygen to mucosal tissues, ensuring their proper function and survival. This exchange is critical for maintaining the health and integrity of mucosal barriers. This review will discuss the OOCs used to represent the mucosal architecture and vasculature, and it can encourage us to think of ways in which the integration of both can better mimic the complexities of biological systems and gain deeper insights into various physiological and pathological processes. This will help to facilitate the development of more accurate predictive models, which are invaluable for advancing our understanding of disease mechanisms and developing novel therapeutic interventions.

## 1. Introduction

Organ-on-chip (OOC) devices are microfluidic devices that do exactly what their name implies: they mimic an organ, but on a chip, which involves the combination of engineering and biology. They have recently become well known in biomedical research due to their ability to replicate the functions and structures of tissues/organs in vivo, which allows researchers to study phenomena that would have been impossible to study using traditional in vitro methods. OOCs address many limitations that traditional 2D and 3D cell-culture techniques have [1]. Many of the disadvantages of 2D cell cultures encompass the idea that these models are not physiologically relevant. Some of these limitations include that the cells do not differentiate very well, cell–cell junctions do not form, drug studies are not possible, cell proliferation is unnatural, and gene expression is not accurate with 2D cell cultures when compared to in vivo models. On the other hand, 3D cell cultures address many of the limitations of the 2D technique, but they still have limitations themselves, like the fact that the matrices/scaffolds used to form them may contain unintended external components, making them less adaptable to clinical setups, and they suffer from batch-to-batch variability.

Organ-on-chip models are the result of merging tissue bioengineering with microfluidic technologies. Microfluidics have played a pivotal role in OOC development by enabling the creation of intricate physical microenvironments that closely resemble physiological systems on a miniature scale. These microarchitectures offer a more faithful representation of natural biological functions outside the body and facilitate high-throughput approaches essential for disease modeling, personalized medicine, and drug testing.

The first reported OOC was fabricated by Huh et al. in 2007, where they developed a lung-on-a-chip that could model excess fluid accumulation occurring in diseased lungs; they were successful in modeling the airways of the lungs [2]. Thus, this organ-on-chip technology reflects a rapidly advancing field that holds great promise for advancing our understanding of human physiology, disease mechanisms, and drug responses, leading to improved healthcare outcomes. In this review, we would like to discuss how mucosa and vasculature have been used to mimic different organs and implemented in different organ-on-chip platforms and their physiological relevance. In OOCs, incorporating mucosal layers helps replicate physiological barriers, and integrating vasculature is crucial for representing the dynamic nutrient and waste exchange processes.. We have picked these two because incorporating these two types of constructs into organ-on-chip models would help to replicate key structural and functional aspects of human organs, enabling more accurate studies of organ physiology, disease mechanisms, drug responses, and toxicity assessments.

## 2. Studying Mechanical Cues in the Epithelial Barrier Using a 3D Oral Epi-Mucosa Platform

The maintenance of oral tissue homeostasis depends on the coordinated function of various cell types, including perivascular, nerve, endothelial, and epithelial cells, as well as their spatial arrangement in three-dimensional space through interactions with the extracellular matrix (ECM). This spatial organization provides biochemical and mechanical cues that are crucial for cellular function. Disruptions in the epithelial barrier’s functioning can lead to an imbalance between the host and surrounding bacteria, resulting in inflammation, gingivitis, and periodontitis, which can cause tissue damage and potential tooth loss. Although the impact of bacteria-induced inflammation on epithelial function is recognized, the role of mechanical cues and tissue microenvironments in regulating barrier function in the oral mucosa remains inadequately understood. Periodontitis causes an increase in interstitial pressure in the oral microenvironment [2,3]. This is accompanied by leaky blood vessels, a five-fold increase in the flow of gingival crevicular fluid, and the formation of a periodontal pocket. To investigate the role of mechanical disruptions in the regulation of oral tissue homeostasis, an experimental system that can mimic the dynamics of the tissue microenvironment of the sulcus would be helpful. Lee et al. developed a microfluidic device that mimics the epithelial barrier of the human oral microenvironment. Their 3D oral epi-mucosa device was used as an experimental model to investigate the role of mechanical disruptions in the development of the oral epithelial barrier.

Specifically, the authors aimed to assess how adjusting the concentrations of collagen I and fibronectin in a 3D fibrous microenvironment would influence the epithelial barrier’s functioning. These two components of the extracellular matrix, fibronectin and E-cadherin, are essential for maintaining the structure and function of gingival tissue. Their study found that the application of mechanical stress enhanced the barrier function and the expression of E-cadherin. Additionally, fibronectin increased epithelial barrier function while decreasing the expression of inter-epithelial adhesion molecules.

**Figure 1:**
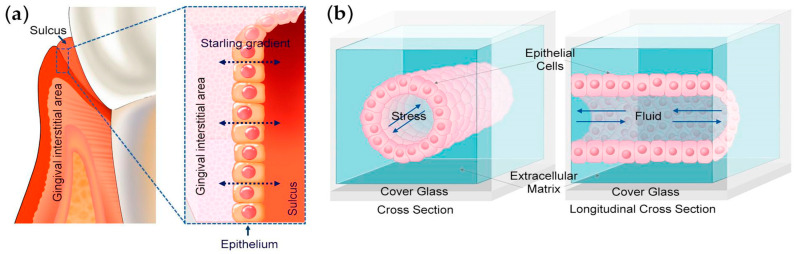
Engineering the 3D oral epi-mucosa platform: (**a**) Starling gradient between the interstitial gingival area and sulcus. (**b**) Schematic of the cross-sectional area of the epi-mucosa-on-a-chip platform. Oral keratinocytes were seeded in a 3D extracellular matrix within a microfabricated PDMS gasket. Creative Common CC BY 4.0 license; Adapted from Ref. [4].

Their study employed a microfluidic device fabricated with a 3D-printed scaffold (Figure 1) [4]. The scaffold, made from poly dimethyl siloxane (PDMS), was treated with glutaraldehyde to promote the adhesion of collagen I. Human immortalized gingival keratinocytes were seeded on the top surface of the channel in the device and then flipped to attach to the bottom channel as well. Applying a low-level interstitial pressure of approximately 0.1 kPa enhanced the epithelial barrier function. This allowed for investigations into the effects of extracellular matrix stiffness on keratinocytes and the impact of fibronectin on the matrix.

Normal gingival tissue primarily contains type I collagen and fibronectin, which provide structural integrity and mechanical strength to the connective tissue. Fibronectin also plays a crucial role in controlling the stiffness of the extracellular matrix, which affects cell adhesion, spreading, and migration [5,6]. The mucosa platform evaluated the effect of the three-dimensional microenvironment on the barrier function by measuring the stiffness and total stress response of the underlying matrix. To investigate the impact of matrix stiffness on cell adhesion molecules, cells were cultured under different collagen concentrations ranging from 1.5 mg/mL to 6 mg/mL. It was discovered that the shear modulus was proportional to the increase in collagen concentration, while the loss of tangent was not affected. These findings suggest that the mechanical properties of the ECM can be altered to adjust the epithelial barrier. Interestingly, the combination of collagen and fibronectin resulted in higher shear modulus and stiffness values compared to collagen alone. Additionally, the cells’ attachment to fibronectin was greater, and the cells exhibited a spindle-like morphology with increased spreading. The addition of fibronectin resulted in a significant increase in epithelial permeability compared to collagen gels, indicating a negative impact on barrier integrity. Interestingly, the absence of mechanical stress caused a 17-fold increase in epithelial permeability for cells in collagen with fibronectin gels, and about a 10-fold increase for cells in collagen-only gels. It was found that the absence of mechanical stress and fibronectin’s presence negatively impacted epithelial integrity. Additionally, matrix stiffness was found to increase the localization of E-cadherin and mRNA levels of inter-epithelial adhesion molecules. However, this was also found to decrease the expression of local adhesion molecules and hemidesmosomes [7].

This OOC platform enables discussions on alterations in the matrix porosity, binding kinetics, stress relaxation in the ECM, active contractile forces, and the mechanical remodeling of the matrix and associated feedback between cells, and how the ECM affects the barrier functions in the oral platform. The chip permits the measurement of epithelial integrity. Moreover, not only can this device recapitulate the mechanical forces resulting from the influence of the extracellular matrix and how it impacts the oral epithelial cells in vivo, but it can also help us to establish the fact that mechanical stress regulates epithelial integrity. The remaining challenges can be addressed by delving into how different cell types influence the characteristics of the surrounding matrix and the function of the epithelial barrier.

Overall, this 3D oral epi-mucosa device facilitates the exploration of critical mechanobiological factors associated with the maintenance of epithelial barrier function. This experimental system could aid in the discovery of new pathways involved in oral diseases and in the discovery of new therapeutics for the treatment of periodontitis [8,9].

## 3. In Vitro OOC Model for Studying the Gingival Crevice

Periodontitis, a chronic inflammatory disease, is primarily caused by bacterial plaque buildup in the gingival crevice, the V-shaped crevice between the tooth and the gingiva. The development of periodontitis involves complex interactions among the plaque microbiome residing within the gingival crevice, the gingival tissues, immune components, and the gingival crevicular fluid (GCF). To replicate the gingival crevice in vitro, a gingival microenvironment would need to be created. This would include gingival tissue cells, the extracellular matrix or scaffold, and the oral microbiome. Studying the interactions between the host and microbiome in periodontal conditions has been challenging due to the limitations of existing in vitro pre-clinical models. Specifically, these models fail to replicate the dynamic nature of the crevicular fluid [10]. To address this issue, most studies rely on exposing gingival and periodontal cells cultured in monolayers on static culture plates to plaque microbes or their substitutes.

In simplified systems, microbes or their substitutes are directly introduced into the culture medium where host cells are cultivated [11,12]. For instance, studies that use co-cultures of human cells with live bacteria often face limitations in investigating host–oral microbe interactions due to the accumulation of toxic by-products from bacterial metabolism that can damage host cells. This interaction between host cells and the live oral microbiome is typically limited to brief time frames, usually ranging from 6 to 24 h [13,14]. Researchers have alternatively used attenuated bacteria or substitutes such as Toll-like receptor (TLR) agonists or virulence factors like lipopolysaccharides (LPSs) and lipoteichoic acid [15]. The role of GCF and its flow in host–microbe interactions has not been taken into consideration in these studies. Establishing a stable symbiotic relationship between the gingival crevicular tissue and the plaque microbiome is essential for studying the initiation and progression of periodontal disease. This relationship occurs in a dynamic environment continuously bathed in GCF. The GCF plays a crucial role in creating conditions that reflect the natural dynamics of periodontal health and diseases. Investigating interactions within a dynamic setting is crucial for gaining insights into the complexities of periodontal disease development and progression. Therefore, innovative methods are needed to create more physiologically relevant in vitro models to better understand the initiation and progression of periodontitis.

**Figure 2:**
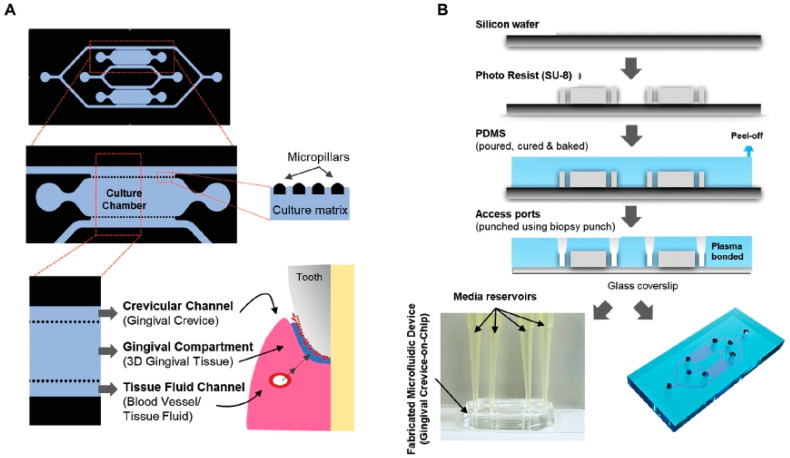
Gingival crevice-on-chip microfluidic device. (**A**) Schematic of the microfluidic device showing the overall design and its various components. (**B**) Schematic showing the fabrication process that utilized soft lithography. Reprinted with permission from Ref. [10], 2022 Wiley-VCH GmbH.

Makkar et al. developed a gingival-crevice-on-chip microfluidic device to study oral host–microbiome interactions in the context of periodontal diseases. This device, shown in Figure 2 [10], has a hexagonal chamber with two fluidic channel inlets: one for gingival crevicular fluid and the other for tissue fluid, represented by the blood supply. The flow of GCF was regulated by adjusting the volumes between the media reservoirs that supplied the tissue fluid, representing the blood supply, and the crevicular channels, which imitate the gingival crevice. The oral microbiome was cultured in the crevicular channel with gingival connective tissue equivalents to mimic the gingival crevice. Micropillars were fabricated to compartmentalize the matrix within the cells, serving as burst valves between the culture chambers and the channels.

This device was created to establish a more realistic microenvironment for studying the mechanisms of healthy and inflamed gingival connective tissues. This can be achieved by exposing the tissues to pathogenic bacteria and TLR 2 agonists. Additionally, the device allows co-cultures with commensal bacteria, enabling the quantification of long-term microbial colonization and biofilm formation. The objective of Makkar et al.’s study was to replicate the characteristics present at the tissue microbiome interface in cases of periodontitis. Additionally, the study examines the protective effect of exposing the gingival crevicular fluid flow towards the bacterial front, rather than direct exposure, which reduces the secretion of inflammatory mediators.

The study replicated the natural flow of gingival crevicular fluid by directing the culture medium unidirectionally through the crevicular channel to the gingival connective tissue equivalents. Human gingival tissues were used to isolate primary connective tissue cells. Streptococcus oralis, an oral commensal bacterium, was introduced to colonize these cells. To facilitate this, the crevicular channel was coated with human saliva overnight before introducing the bacteria. To ensure objectivity, the initial setup remained static. After 8 h, interstitial flow from the tissue fluid channel to the crevicular channel was initiated. The bacteria became attached to the crevicular channel and interfaced with the connective tissue matrix between the micropillars. Following 24 h of co-culturing, the bacteria formed biofilm-like structures, with some bacteria penetrating the superficial layer of the gingival connective tissue. Lactate dehydrogenase levels increased after five days of co-culturing, indicating that the introduction of s-GCF flow allowed the gingival connective tissue to be cultured after introducing bacteria without compromising the host viability. The provided viability data only represents the co-culture conditions and not the bacteria alone. The flow resulted in a three-fold reduction in bacterial load in the crevicular channel and allowed for long-term co-cultures.

To investigate the role of non-commensal microbes in gingival inflammation, the gingival connective tissue equivalents were exposed to a Toll-like receptor-2 agonist and Gram-negative pathogenic bacteria carrying endotoxins, with and without the influence of s-GCF flow. During static conditions or when there was no flow, several inflammatory cytokines, including IL-6, IL-8, and CCL-2, were significantly upregulated. These cytokines were downregulated upon restoration of the s-GCF flow, indicating that stimulatory gingival crevicular fluid flow plays a significant role in controlling the immune response of the gingival tissue. In summary, these findings suggest that this gingival-crevice-on-chip device, due to its ability to facilitate unidirectional interstitial flow, can effectively separate host tissues from a bacterial presence. Additionally, it allows for the in vitro modeling of microbial colonization, followed by the clearance of microbes and mitigation of the innate immune response. This device could be used to study how the extracellular matrix influences microbial colonization in the presence and absence of s-GCF flow.

Although the platform did not support co-culturing for a longer time due to a sudden increase in oxidative stress caused by the biofilm formation from the streptococci, this approach allowed for the replication of microbial colonization, subsequent microbial elimination, and the dampening of the innate immune response triggered by gingival crevicular fluid flow, all within an in vitro setting.

Another recent study aimed to address issues related to chemotherapy- and radiotherapy-induced mucositis by using a microfluidic device. This device mimicked the relevant conditions by co-culturing fibroblasts and endothelial cells in rat-tail collagen, using keratinocytes as a monolayer in the adjacent fluidic channel. The gingival-crevice-on-chip device can be changed and improvised using similar strategies to develop more insights into studying the microbiome’s interactions with the gingival crevice. This crevice-on-a-chip model can be used to explore the effects of oral systems and develop novel anti-inflammatory strategies for periodontal therapy. These improvements will help us to understand long-term host–oral microbiome interactions in healthy and diseased periodontal tissues.

## 4. Biomimicking the Gastric Environment of a Human Stomach Using OOCs for Drug Testing

The current in vitro approaches and animal models are not able to mimic human gastrointestinal diseases like peptic ulcers and gastric cancers because they have certain limitations, including being unable to represent the complex human gastrointestinal physiology or mimic the dynamic nature of the gastric mucosa. To create a more physiologically relevant environment, some authors have used tissue engineering approach to produce a stomach-system-on-a-chip microdevice. The microfluidic stomach-on-a-chip device created by Ferreira et al. mimics the peristalsis-like motion of the stomach and intraluminal flow, closely resembling the functional part of stomach, and aims to represent the three layers of the stomach wall, i.e., the gastric mucosa, consisting of the epithelium, the basement membrane, and the lamina propria.

**Figure 3:**
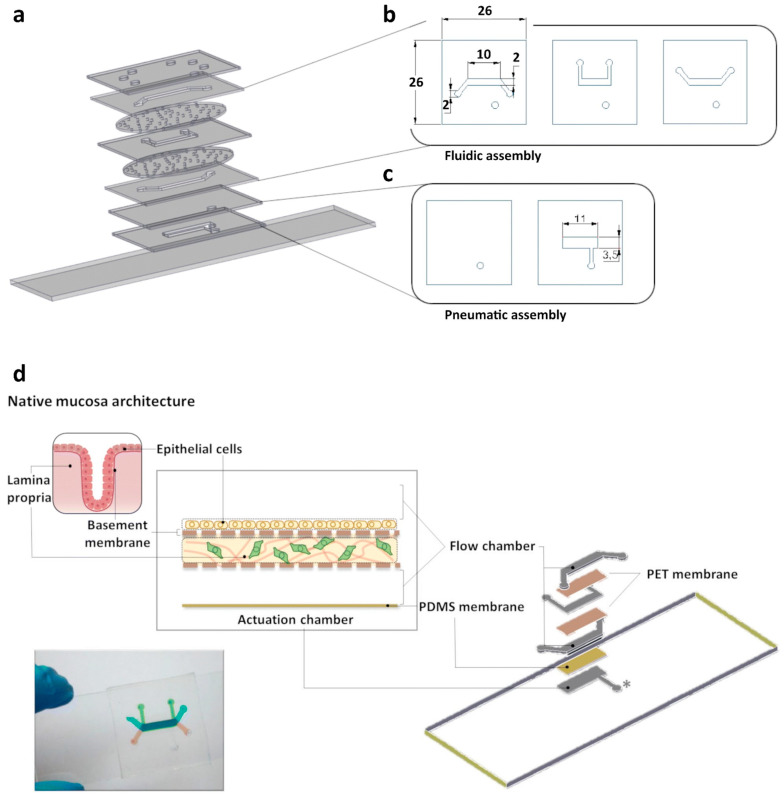
Schematic of the stomach-on-a-chip device, highlighting the engineered gastric mucosa and the three innermost layers of the gastric wall, namely the epithelial barrier, the basement membrane, and the lamina propria. (**a**) Exploded view of the SoC depicting the 9 intercalating parts composing the fluidic and pneumatic assembly: the topmost layer corresponds to a thick PDMS layer produced by gravity casting to support tube attachment, while the bottommost layer corresponds to a microscope glass slide. (**b**) Top-down view of the fluidic structures composing the fluidic assembly. Each of the layers is intercalated by a PET membrane. (**c**) Top down view of the 2-part pneumatic assembly consisting of a thin 250 μm PDMS membrane (left) and a vacuum chamber placed underneath the membrane (right). (**d**) Schematic of the SoC, highlighting the engineered gastric mucosa with a graphical representation of the 3 innermost layers of the gastric wall, namely the epithelial barrier, the basement membrane and the lamina propria. The exploded view of the biochip, depicts the fluidic and microactuator (*) structures in grey. Creative Commons Attribution Non-Commercial 3.0 Unported license; Reprinted from Ref. [16].

To represent the three-layered epithelial interface, their research designed a multilayered microfluidic device, as shown in Figure 3 [16]. The top layer was produced from a thick layer of PDMS, fabricated through gravity casting, while the underlying fluidic network was produced from PDMS intercalated with a thick polyethylene terephthalate (PET) membrane. The three-tiered fluidic structure was designed to enable the replication of an epithelial barrier architecture on the top chamber and, importantly, to provide the efficient incorporation of a lamina propria analog in the middle culture chamber. A reconstituted basement membrane was positioned as the bottom layer. The lamina propria analog, composed of collagen and gastric fibroblasts, can enhance communication between the lamina propria and the epithelial layer through paracrine signaling [16]. The entire chip was sealed against a glass slide.

The MKN74 epithelial gastric cell line was used because in order to replicate the selective permeability of the stomach, a tightly packed monolayer of cells was required, wherein the cells should display tight cell contacts between themselves. This cell line displays moderate intestinal differentiation, including cell polarity and the presence of microvilli with core filaments and several characteristics relating to its epithelial origin, displaying localized tight junctions that mediate paracellular traffic across the epithelial barrier, as shown with ZO-1 and Occludin staining [16]. Trans-epithelial measurements were made to assess the ability of the cells to form a tight barrier. Collagen I gel was used to form the lamina propria as it the most prevalent extracellular matrix protein found there [17]. The normal stomach NST-20 fibroblast cell line was then embedded with collagen to investigate whether they were able to maintain growing conditions and stability in the hydrogel. The authors found an increase in the metabolic activity or cell viability upon an increase in the matrix density, quantified using a resazurin assay. Another study was then performed to find out the effects of ECM gel contraction on the fibroblast-embedded hydrogels.

Here, the authors tried to reconstitute the analog of the anchoring substrate as the basement membrane that anchors the epithelial layer of the stomach in vivo [18,19]. It was found that the MKN74 cell line tried to form a sheet-like epithelial monolayer and displayed a typical epithelial-cell-like phenotype when seeded in pure fibronectin gel, and hence fibronectin was able to mimic the adhesion characteristics of the membrane and helped in the formation of a cell monolayer under flow conditions. The tightly formed epithelial layer was verified with E-cadherin and ZO-1 staining, which confirmed the formation of fully functional and tight cell-to-cell contacts [16]. Pepsin activity assays were conducted between static and dynamic culture conditions and compared. It was found that the mechanical stretching was able to affect the conversion of pepsin from pepsinogen, as occurs in vivo [16]. To assess selective permeability to small molecules and drugs, which is another important characteristic displayed by the human stomach, fluorescein isothiocyanate labelled dextran was used as a transport molecule across the epithelium under certain experimental conditions. A prominent difference was observed in paracellular permeability for the cells growing under static conditions compared to those growing under dynamic conditions, with a significant difference already being observable at 30 min after perfusion. The induction of flow was sufficient to cause a marked decrease in the gastric epithelium’s permeability, making it more established that dynamic conditions are much more important for mimicking the gastric environment in vivo. Additionally, the peristalsis-like movement and simulation of intra-luminal flow generated stimuli that were physiologically significant. This stimulation prompted the MKN74 cell line, which is typically moderately differentiated and tends to form a squamous-like epithelium, to undergo further differentiation. As a result, the cells acquired phenotypical characteristics commonly observed in the normal gastric epithelium.

The device mimicked the structure of the gastric mucosa with remarkable accuracy and demonstrated the ability to partially replicate gastric functions. This was evidenced by an improvement in barrier function, indicating a promising step towards replicating the functionalities of the stomach. In the future, incorporating an endothelial layer would help to represent the vasculature and would make the model more suitable to study tumor infiltration and penetration.

The stomach-on-chip device reported here represents a multifaceted model allowing access to the apical and basal structures of the stomach epithelial cells, unlike the organoid-on-chip gastric system previously developed by Lee et al. [20]. This opens up the possibility of investigating other components within the lamina propria, like the immune system and the endothelial interface, to examine how they respond to inflammation, tumor onset, and invasion. Integrating these elements would increase the system’s intricacy and provide a platform for studying diverse aspects of tissue behavior.

## 5. Studying Blood Vascular Tumors Using Versatile Vessel-on-a-Chip Platforms

Vascular tumors cause a wide range of symptoms, varying from minor tissue abnormalities and persistent discomfort to severe and potentially fatal issues like coagulation problems, such as blood clot formation or hemorrhaging. Previously reported in vitro conventional models do exist, but they have many associated issues and limitations such as the inability to create a dynamic microenvironment. Notably, the absence of established therapeutic guidelines for these tumors is, in part, attributable to the limited availability of technological platforms capable of accurately replicating the unique features of the pathology in a straightforward and controlled manner. In addressing this challenge, models like vessel-on-a-chip devices offer a realistic and dynamic microenvironment like the one associated with vascular tumors. Microfluidic technology has shown the potential to monitor blood flow in smaller vessels. Microfluidics-based technology offers significant advantages in regulating physiological flow levels, shear stress, cell content, and chip architecture [21,22]. Vessel-on-a-chip models, extensively documented for investigating blood vessel development, function, and pathogenesis, have commonly utilized standard replica molding and soft lithography to replicate the native geometry of microvascular structures, owing to their simplicity [23]. Lleneas et al. have discussed the creation of a simplified vessel-on-a-chip model for studying the unique characteristics of infantile vascular tumors [24].

**Figure 4:**
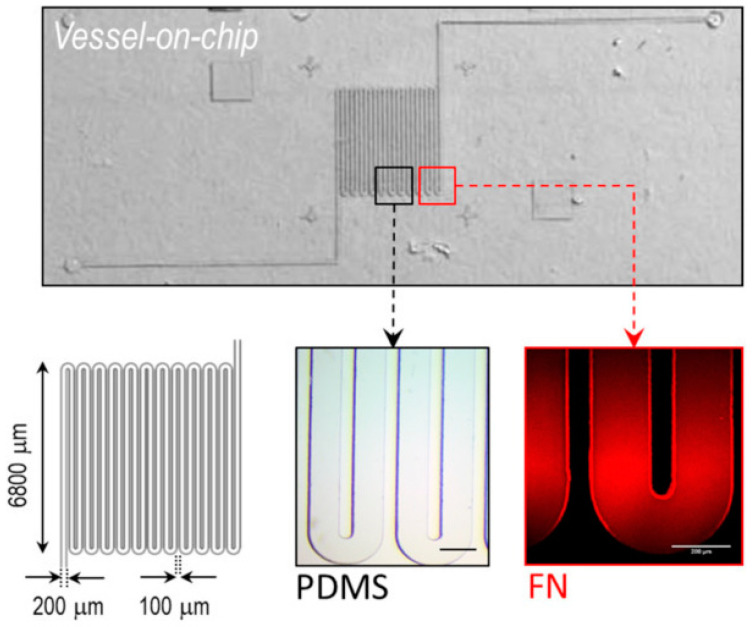
Microfluidic vessel-on-a-chip model. (**Upper**) Optical microscopy image of the polydimethylsiloxane microfluidic chip. (**Lower**) Left—dimensions of the microchannel; middle and right—magnified image of the fabricated and fibronectin-coated (rhodamine) channel. Reproduced under the Creative Common CC BY 4.0 license Reprinted from Ref. [24].

Their model, displayed in Figure 4 [24], consists of a microfluidic channel using standard soft lithography techniques with a serpentine morphology and a rectangular cross section to mimic the architecture of blood flow through a tumor.

In a healthy vascular system, the endothelium naturally prevents blood clotting. However, in pathological conditions, vascular tumors are formed when the blood flow becomes disturbed due to the formation of blood clots. In Lleneas et al.’s research, the vessel-on-chip device was made to mimic the conditions of blood clot formation and hemorrhages to explore the characteristics and features of the tumor. The researchers replicated this condition by replacing the endothelial layer in their vessel-on-a-chip device with a fibronectin coating that activates clot formation. The chip was then perfused with diluted human blood at a low flow rate, which quickly led to the formation of blood-clot-like aggregates.

In silico simulations showed a parabolic velocity profile characteristic of blood flow. The viscosity of the blood exhibited a similar pattern in the linear channel regions but increased at the bends of the serpentine microchannel. Experimental measurements of blood hydrodynamics were conducted using fluorescent beads to track the velocity profiles of the red blood cells. A large scattering of red blood cells indicated that the hematocrit percentage was high. The utilization of this phenomenon can be harnessed via drug-loaded nanocarriers to achieve effective accumulation in the intended region. In particular, the directed movement of these carriers towards the vessel walls can enhance their ability to traverse the fenestrated endothelium commonly found in tumoral endothelia [25].

In a healthy and normal vascular system, the endothelium possesses a non-adhesive quality, contributing to a natural anti-thrombotic effect that facilitates the proper transport of red blood cells (RBCs). However, under pathological conditions, the endothelium undergoes changes in the local expression and secretion of adhesive proteins, promoting the formation of thrombi. To replicate this condition in an experimental setup, Lleneas et al. replaced the endothelial layer with a clot-activating fibronectin coating, considering that the blood contained the anti-coagulant ethylenediaminetetraacetic acid (EDTA) to prevent clot formation. For this experiment, a reductionistic vessel-on-a-chip device was utilized, wherein diluted human blood was perfused at a constant low flow rate. This approach aimed to encourage the adhesion of RBCs, leading to the rapid and substantial formation of blood-clot-like aggregates. Future enhancements to this research may involve integrating a complete endothelium and employing non-treated whole blood. The number and size of the aggregates increased over time, ranging from small clusters to larger blood clots that spanned the width of the channel. The researchers noted a significant rise in blood velocity within a sizable thrombus, a phenomenon they also observed in the simulations. This heightened velocity was attributed to the non-Newtonian characteristics of the blood, marked by shear thinning and reduced viscosity. The disturbed flow further induced a stochastic rolling of RBCs, as documented in previous studies [26]. Subsequently, an exploration into the hydrodynamics of RBCs in regions proximate to thrombus formation was conducted using real-time optical microscopy and label-free micro-particle image velocimetry (micro-PIV). This analysis revealed an anticipated velocity field wherein the velocity of the RBCs was higher in the constricted regions.

Nonetheless, because there was no actual endothelial layer present, assumptions were made regarding the formation of the blood clots, which may have been caused by direct platelet activation and fibrin buildup, replicating the natural coagulation process. To substantiate this, further refinement is required to investigate the precise mechanisms and molecular attributes of clot formation. This would entail introducing endothelial cells and using untreated blood, as some studies utilizing microfluidics have demonstrated the essential pro-coagulant role of the endothelium in hemostasis [27]. To enhance the physiological relevance of the platform, several improvements can be made by (1) integrating both thrombus- and hemorrhage-on-a-chip models lined with endothelial cells to enable the measurement of key clinical parameters such as the bleeding time, the assessment of (anti-) coagulant formulations, or the evaluation of health and coverage under pathological conditions; (2) updating the geometry of the microchannels to better mimic native vessel morphology; (3) using a hydrogel instead of solid PDMS to better replicate the native microenvironment [28]; and also (4) utilizing fresh blood without anti-coagulants to analyze the difference. These enhancements would provide a better understanding of blood flow phenomena, contributing to the improved prevention, diagnosis, and treatment of vascular diseases, particularly in the context of infantile vascular tumors.

## 6. Utilizing a Self-Assembled Innervated Vasculature-on-a-Chip Device to Study Nociception

Interactions between the vascular system and sensory neurons are vital for understanding the mechanisms behind inflammatory pain. Inflammatory cytokines play a significant role in stimulating sensory neurons and enhancing pain sensitivity. Nociception plays a crucial role in safeguarding tissues from harm and facilitating the healing process following injuries. Sensory neurons extensively populate peripheral tissues, detecting and reacting to harmful mechanical, thermal, and chemical stimuli. Nociceptor afferents are equipped with ligand- and voltage-gated ion channels, serving as essential molecular transducers for noxious stimuli [29]. Another chemically harmful stimulus of clinical significance is linked to tissue acidosis, a state marked by an elevated concentration of protons, indicated by a low pH [30]. Tissue acidosis is correlated with various pathological conditions, including cancer, respiratory diseases, and muscular disorders [30,31,32].

The absence of in vitro models that accurately mimic physiological conditions poses a substantial obstacle in comprehending the mechanisms behind these conditions and developing new drugs, including analgesics, to alleviate pain [33].

**Figure 5:**
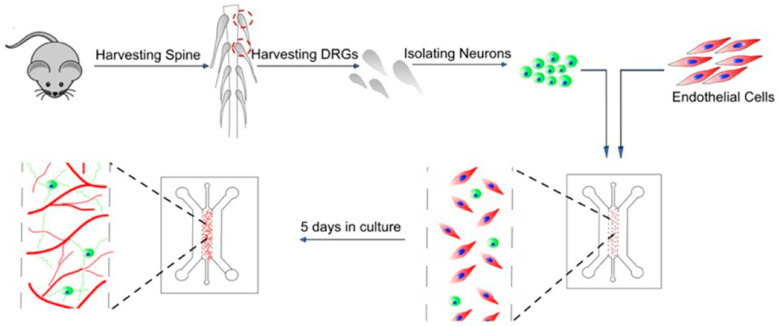
Schematic of the cell isolation and process of generating innervated vasculature tissue by co-culturing DRG neurons and endothelial cells. Reprinted with permission from Ref. [29], 2023 Kumar et al.

Here, to study nociception in organ on chip, a microfluidic model of innervated vasculature was developed to explore the interaction between sensory neurons and the vascular system. Co-culturing human umbilical vein endothelial cells (HUVECs) with primary dorsal root ganglion (DRG) neurons collected from both mice and human donors resulted in the creation of perfusable vascular networks that became innervated by a three-dimensional network of neurites originating from the sensory neurons. These neurites intricately surrounded the vessels, establishing direct contact with the endothelial cells and facilitating the activation of sensory neurons in response to chemical stimuli in the microvasculature. By utilizing this platform, the researchers also investigated the impact of acidosis on sensory neurons by reducing the pH of the perfusate within the microvasculature. The poly dimethyl siloxane (PDMS) device employed in this study, shown in Figure 5 [29], has been utilized in various research studies, including investigations into vasculature [34,35], the blood–brain barrier [34,35,36], and tumors [37]. In research involving the simultaneous cultivation of DRG neurons and endothelial cells without direct interaction, an additional set of hydrogel and media microchannels was incorporated into the design. This modification ensured that the hydrogel microchannels, housing sensory neurons and endothelial cells, were kept apart by the media microchannel. This spatial division avoids direct cell contact while enabling communication between cells through the exchange of secreted factors.

The primary types of neurons that innervate blood vessels are sympathetic, parasympathetic, and sensory neurons. Sympathetic neurons governing blood vessels are responsible for constriction, while sensory neurons contribute to vasodilation by releasing neuropeptides and neurotransmitters. Several clinical conditions, such as diabetic neuropathy, Raynaud’s disease, and fibromyalgia, are linked to dysfunctional neuronal activity within the vascular system.

Co-cultures of human umbilical vein endothelial cells (HUVECs) and dorsal root ganglia (DRG) neurons were performed for the device as shown in Figure 5. To examine the impact of co-culturing on each cell type, mouse DRG neurons and endothelial cells were subjected to four distinct conditions: (i) a monoculture of DRG neurons, (ii) a monoculture of HUVECs, (iii) a co-culture of DRG neurons and HUVECs without direct cell–cell contact, and (iv) a co-culture of DRG neurons and HUVECs with direct cell–cell contact. The evaluation of neurite formation aimed to elucidate the influence of endothelial cells on sensory neurons, while the quantification of vessel structure aimed to assess the impact of sensory neurons on vasculature. In the non-contact co-culture experiment, there was a significant increase in the percentage of DRG neurons with sprouted neurites, as well as in the number and length of neurites, compared to the monoculture of DRG neurons. When comparing the two co-culture conditions, direct cell–cell contact resulted in a significantly greater number and length of neurites. Like the effect of vascular cells on neurons, the co-culture results also demonstrated a notable difference in the vascular network. Specifically, the vessel diameter was significantly higher when the endothelial cells were co-cultured with DRG neurons in direct contact compared to the monoculture of endothelial cells. No differences were observed in vascular branch density among the three conditions, but the area covered by the vasculature was significantly higher under the direct-contact co-culture conditions.

The data indicated that the existence of endothelial cells heightens the sensitivity of sensory neurons, rendering them more responsive to chemical stimuli like capsaicin, as seen by an elevated expression of the capsaicin receptor TRPV1. The functionality of DRG neurons persisted within the innervated vascular tissue, establishing them as a viable platform for investigating acid nociception.

Given the more pronounced differences in neuronal and vascular phenotypes observed in the direct contact co-culture system, this approach was selected for subsequent experiments and was compared against the monoculture of DRG neurons. The platform was also utilized to model nociception related to acid sensing during acidosis. As the pH of the vascular perfusate decreased, the response of the sensory neurons increased, like the sensitivity to acidosis seen in different vascular diseases and pain-related conditions.

The study also verified the platform’s capability to support the cultivation of primary DRG neurons sourced from human clinical specimens. Human primary DRGs, obtained from patients who consented to the procedure of spinal decompression and fusion for metastatic epidural compression, underwent dissociation, and the cultured DRG neurons were combined with endothelial cells within the device. Although no disparity in neurite length was noted between the monoculture and co-culture of sensory neurons, the co-culture exhibited a significantly higher number of neurites per cell. Also, the expression of TRPV1 on the sensory neurons, determined through immunostaining, was higher in the presence of microvasculature. These results align with those observed in murine DRG neurons, indicating an increased level of sensitization in the presence of vasculature.

The study investigated the neurons’ responsiveness to noxious stimuli within the innervated vasculature, using capsaicin and tissue acidosis as model systems. Devices of this nature can prove invaluable for understanding the molecular mechanisms underlying pain and can also function as platforms for testing therapeutics and analgesics. In addition to utilizing the perfusable vascular network to examine the impact of bloodborne factors on nociception, microfluidic-assisted micro physiological systems are well-positioned to integrate dynamic mechanical cues [38,39,40,41], as well as vascular-specific functions such as vasodilation. All of these microenvironmental cues play a crucial role in nociception [42,43].

This study introduces the development of a novel innervated vascular tissue model on a chip. This model involves co-culturing endothelial cells and DRG neurons within a microfluidic device. Within this setup, the endothelial cells naturally organize into functional microvascular networks, while the DRG neurons extend neurites to innervate these networks. Interestingly, the interaction between these endothelial cells and DRG neurons enhances both the vascular lumens and neurite density compared to cultures of each cell type alone. Specifically, direct contact between DRG neurons and endothelial cells promotes greater neurite growth in quantity and length. These findings align with previous studies involving co-cultures of endothelial cells and motor neurons. The observed differences in cellular behavior between co-cultures with and without direct contact may stem from alterations in secretory factors or variations in the diffusion and stability of these molecules within the microenvironment.

To further confirm the results, primary sensory neurons obtained from human donors were employed, establishing a model of innervated vasculature specific to humans. This holds significance due to the variations between mouse and human sensory neurons, and utilizing human models offers a more precise understanding of nociceptive mechanisms. The use of this organ-on-a-chip (OOC) device can advance our comprehension of nociception and contribute to the creation of precise interventions for conditions related to pain.

## 7. Utilizing OOCs to Investigate Biomechanical Characteristics in Tissue-Engineered Blood Vessels

Cardiovascular diseases are the leading cause of death, not only in the US but worldwide. Certain cardiovascular diseases can be diagnosed clinically by replacing damaged blood vessels with alternative substitutes, such as tissue-engineered blood vessels (TEBVs). Various artificial blood vessels have been created using synthetic polymers, rubber, hydrogels, and other materials. However, these substitutes have limitations in relation to biological and physiological functions, even though their mechanical stiffness and strength are similar to those of native blood vessels. Significant endeavors have been made in the development of different artificial blood vessels and blood-vessel-on-a-chip models, employing the tissue-engineered approach [44,45]. Tissue-engineered blood vessels (TEBVs) created from synthetic polymers, polyglycolic acid, or L-lactide were among the first to be reported. Efforts have also led to the development of small-diameter TEBVs using collagen scaffolds seeded with human umbilical cord blood-derived endothelial progenitor cells and umbilical artery smooth muscle cells [46]. TEBVs hold great promise as a technology for creating artificial blood vessels that mimic the properties of natural vessels, including their biological, biochemical, and biomechanical characteristics. They offer several advantages over traditional artificial vessels, including preventing blood clotting through endothelialization, enhancing biological functions by seeding with smooth muscle cells, and providing appropriate biomechanical strength and stress–strain relationships.

**Figure 6:**
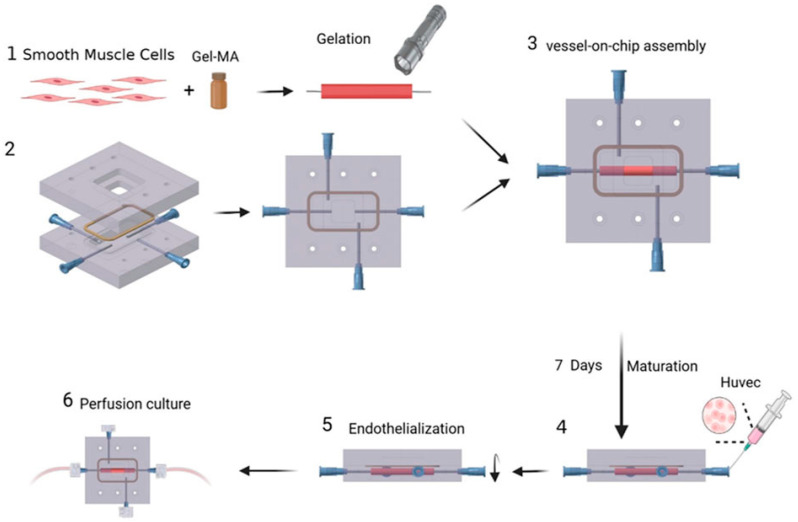
Schematic showing the construction of the TEBV-on-a-chip device. 1. Human aortic smooth muscle cells were embedded in gelatin methacryloyl (GelMA) and the mixture was injected into a cylindrical mold with a mandrel in the middle for gelation. 2. The microfluidic device dabrication. 3. After gelation, TEBV was placed into the device to construct the blood vessel-on-a-chip. 4. TEBV in the chip was supplied with smooth muscle cell media to mature for 1 week. After that, HUVECs were injected onto the inner surface of TEBV. 5. The formation of functiona l endothelium takes place. 6. The vessel lumen of TEBV was connected to two perfusion ports as the inlet and outlet for the perfusion. Creative Common Attributions license (CC BY). Reprinted from Ref. [47].

The vessel construct represented in Figure 6 [47] consists of human aortic smooth muscle cells(HASMCs) and is seeded with human umbilical vein endothelial cells(HUVEC) cells on its inner surface. Hematoxylin and eosin (H&E) staining on frozen sections was conducted to verify the presence of these cells. Using poly dimethly siloxane(PDMS), a microfluidic device with a gel-molding chamber and a perfusion chamber was fabricated. The TEBV was positioned within the gel-molding chamber to create the TEBV-on-a-chip model, and was then secured with surgical suturing. The vessel lumen of the TEBV was linked to two perfusion ports, serving as the inlet and outlet for the perfusion flow. The viscosity of the cell culture media was measured to analyze the data between the shear stress and strain.

Fluid–structure interaction (FSI) modeling was utilized to analyze the biomechanical environment within the walls of a TEBV and fluid flow [47]. The vascular construct for the TEBV was created in a cylindrical form, following a method described by Lee et al. [48]. HASMCs were embedded within a gelatin meth acryloyl (GelMA) hydrogel, and this cell–gel mixture was then injected into a cylindrical mold containing a central mandrel for support during gelation. After a 1 min gelation period using blue visible light, the TEBV was inserted into a microfluidic device made of PDMS to establish a blood-vessel-on-a-chip system. The HUVECs were then injected to after maturation for 1 week to form a functional endothelium and then the device was perfused with culture media.

This FSI modeling study fills a gap in our understanding of biomechanics in tissue-engineered organs by offering quantitative insights into the mechanical conditions within these systems. It uses tissue-engineered organs as a modeling approach to investigate biomechanics and its impact on the functional properties of these organs.

A comparison study was also conducted between the behavior of tissue-engineered blood vessels (TEBVs) during a perfusion experiment and the results obtained from a fluid–structure interaction (FSI) simulation. Overall, the analysis showed that both the TEBV movement and fluid dynamics in the experiment and the FSI model were comparable.

Existing evidence indicates that these structural mechanics are crucial for optimizing the biological functions of organ-on-chip technology, particularly in models like lung-on-a-chip and heart-on-a-chip devices [49,50]. Mechanical stimulations have been shown to enhance cellular behaviors and cell–cell or cell–scaffold interactions, better mimicking the physiological function of organs. This study represents the first fluid–structure interaction modeling effort to quantitatively capture the mechanical conditions in tissue-engineered organs. Leveraging tissue-engineered organs as a modeling basis, this approach stands as a potent tool for investigating biomechanics and studying its impact on the functional properties of tissue-engineered organs.

The biochemical conditions of cardiovascular diseases, such as strokes or heart attacks, play a critical role in the development and rupture of atherosclerosis in the carotid and coronary arteries. Many studies in this field use clinical images from patients to simulate biomechanical conditions and investigate the connection between plaque behavior and biomechanics [51]. However, conducting in vivo clinical studies has its limitations. Gathering enough representative clinical cases to fully understand plaque ruptures in humans is challenging. Animal models often do not accurately replicate human arterial atherosclerosis.

The use of TEBV-based perfusion experiments is advantageous because it enables the creation of controlled biomechanical environments for studying their impact on disease progression. It is possible to achieve different biomechanical conditions by adjusting parameters such as the flow rate, TEBV stiffness, or outlet pressure conditions. Combining in vitro experiments with tissue-engineered organs and computational models is a valuable approach for studying the impact of biomechanics on the development, progression, and rupture of atherosclerotic plaques and other related cardiovascular diseases.

## 8. Investigation of Epithelial Wounds on the Cornea Using a Cornea-on-a-Chip Device

Organs-on-chips are microfluidic devices designed for cell culturing that simulate tissue-level or organ-level physiology while recapitulating their microenvironment. These platforms offer alternative and impactful solutions to traditional animal testing. In ocular biological studies, in vitro testing platforms have gained increased prominence for their preclinical efficacy and better therapeutic designs. Yu et al. developed a microfluidic platform in their study that incorporates human corneal cells and a porous membrane to replicate the multi-scale structural organization and biological phenotype of the cornea [52].

Although 2D culture models provide cost-effective data, they do not fully capture the complex pathophysiology observed in patients and often fail to predict in vivo responses. According to Esch et al. [53], conventional trans-well 3D culture in vitro models offer a more accurate mimicry of physiological barrier function compared to 2D models. However, animal models typically operate under static conditions and diverge from the dynamic conditions observed in vivo, such as eyelid opening and tear film production [53]. Although animal models can replicate physiological complexity at the organism level, ethical concerns persist. Furthermore, studies have demonstrated the limited capacity of these tests to faithfully reproduce the intricate structure of the human cornea due to species-related differences. For example, rabbits have a lower blinking rate, which may cause the drug to interact with the ocular surface for a longer time. This can result in increased drug permeation and potentially affect the preclinical test results [54].

**Figure 7:**
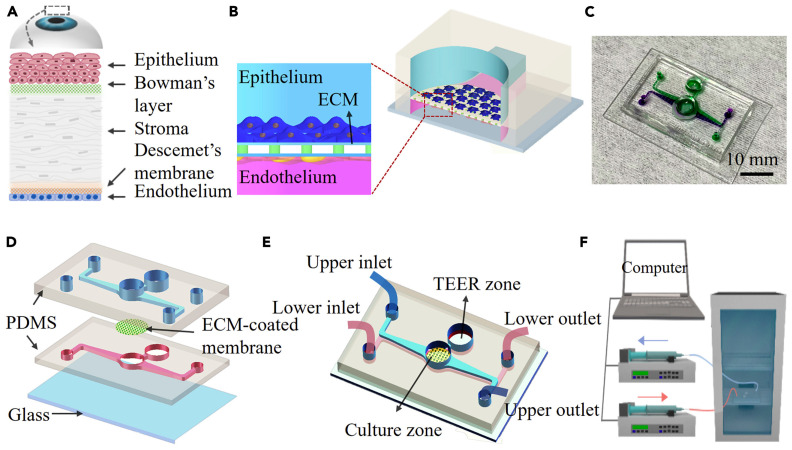
Schematic showing the different layers of the human cornea and design of the cornea-on-a-chip device. (**A**) The anatomy of the human cornea. (**B**) Cross-sectional schematic diagram of the corneal chip. HCEpi and HCEnd cells are cultured on the opposite sides of a porous PC membrane coated with ECM. The membrane is sandwiched between two PDMS layers incorporated with microfluidic channels. (**C**) Photographs of the human cornea-chip; upper and lower microfluidic structures are indicated by using solutions with purple and green color dye. (**D**) Three-dimensional illustration on the device, showing the details on the structures on different layers. (**E**) The cornea-chip with two individual microfluidic channels and a circular hole in the middle mimics the human corneal structure. Structures for in situ TEER measurements are also fabricated on the chip. (**F**) Experimental setup for flow control and cell culture. The device is located in an incubator with two microchannels connected to respective syringe pumps. Creative Commons CC-BY-NC-ND license; Reprinted from Ref. [52].

The microfluidic device shown in Figure 7 [52] was fabricated by bonding a glass substrate with two PDMS layers, with an ECM-coated polycarbonate membrane sandwiched in between. A circular hole was punched in the middle of the microfluidic channel on each PDMS slab to mimic the human corneal structure. The open-top surface of this area was used to create an air–liquid interface and mimic the ocular surface more precisely. The polycarbonate membrane was sterilized using UV germicidal irradiation and then coated with collagen to create an extracellular matrix. Human corneal epithelial (HCEpi) cells were seeded in the upper microchannel, while human corneal endothelial (HCEnd) cells were seeded in the lower microchannel. The measured trans-epithelial electrical resistance (TEER) values were higher as a result, indicating an improved barrier integrity. The HCEpi cells were cultured in trans-well inserts to evaluate the chip’s suitability for corneal cell cultures.

Two experimental setups were created to optimize the culture conditions to mimic the in vivo environment: one with cells grown under immersed conditions and the other with cells grown at the liquid interface. The human corneal cells were maintained in these groups for up to two weeks to assess the microfluidic chip’s suitability for human corneal cell cultures. The cells cultured at the interface showed a better resemblance to the corneal epithelium than those cultured in immersion. The apparent permeability index was used to assess the permeability of the corneal tissue on the chip. The results suggested that the single-cell-type model did not achieve the desired level of permeability, but the co-culture conditions with HCEnd cells improved the barrier’s integrity.

The co-cultured chip demonstrated a gradual increase in TEER values over time and low permeability, indicating the development of corneal tissue. These findings suggest that the microfluidic device, particularly when co-cultured with HCEnd cells, replicates the physiological conditions of the cornea, including maintaining barrier integrity and low permeability. Therefore, it is a promising model for cornea-related research and testing.

The potential of cell-free therapy, specifically extracellular vesicles isolated from the culture media of multipotent stem cells, to heal corneal scratch wounds was demonstrated in the cornea on chip device. This technology shows promise for developing innovative therapeutic approaches for healing corneal scratch wounds in vitro. The corneal wound-healing response is typically triggered by injuries to the epithelium and/or endothelium, which may involve the stroma as well [55]. In cases of mild injuries or infections, such as epithelial abrasions or controlled microbial infections, where there is limited keratocyte apoptosis, the epithelium or endothelium can regenerate, and the epithelial basement membrane can undergo repair. However, in more severe injuries, where there is extensive damage to these membranes, the stroma layer becomes crucial in the wound repair process. Interleukin 1α (IL-1α) is released from the injured epithelium into the stroma. This induces some stromal keratocytes to undergo cell death, while others are stimulated to proliferate and transition from a quiescent to an activated phenotype. The repair of the epithelial basement membrane can eventually lead to a partial resolution of scarring fibrosis [55]. Therefore, the current chip model is suitable for studying mild injuries or infections. However, optimization may be necessary for more severe injuries. Additionally, it would be useful to extend this research to elucidate the mechanisms by which MSC-EVs contribute to the healing of human corneal wounds.

## 9. Conclusions and Future Perspectives

The above OOCs are shaping the way we understand human physiology and its diseased states, especially for mucosal tissues and vasculature. Since these devices are engineered to mimic specific functions and structures, they can be used to model several different tissues and organs. Eventually, many of these OOCs can be connected to form an integrated one, which can be used to model entire organs or parts of the body. This will further extend the application of OOCs, moving away from the traditional in vitro cell culture models and animal models towards a new path that mimics larger functional units of the body.

In this review, we have covered OOCs that represent mucosal tissues and vasculature. These two organ types can be integrated to enhance the representation of a particular mucosal tissue/organ. Mucosa acts as a protective barrier in organs such as the gastrointestinal tract, respiratory tract, vision system, and reproductive organs. This barrier selectively allows the passage of certain molecules while blocking others. In vivo, blood vessels lie beneath the mucosal layer, regulating the transport of nutrients, gases, and waste products. Integrating vasculature into organ-on-chip models beneath the mucosal layer helps mimic this physiological barrier function. It enables the study of how molecules pass through the mucosa, interact with blood vessels, and are transported throughout the body. This is crucial for drug absorption studies, toxicology assessments, and understanding disease mechanisms. The integration of mucosal layers and vasculature in organ-on-chip models would allow researchers to replicate more accurately the physiological complexity and functionality of the mucosal tissues and organs.

Whether they are used in drug development, including for personalized medicine purposes, or in studying inadequately understood phenomena in the body, these devices have already proven to be valuable and will only continue to advance the field of biomedical research. However, they do have certain limitations that should be addressed. The primary barrier hindering the widespread adoption of organ-on-chip technology among commercial or academic users hinges on the quality and quantity of biological data. Numerous OOCs face challenges in terms of their robustness, user-friendliness, and throughput, hindering their ability to produce the extensive and dependable biological data necessary for validation. The absence of online analysis techniques presents a significant obstacle, impeding the real-time monitoring of cultured cells [56]. One notable drawback is the prevalence of surface effects, which become particularly pronounced due to the small dimensions of the fluids, overshadowing volume effects [57]. This can result in a subpar analysis quality and the potential adsorption of the desired product. Additionally, the presence of laminar flow at the juncture of multiple fluids may hinder the proper mixing of the relevant substances [57]. Balancing the requirements for experimental conditions that accurately mirror physiological processes with technical feasibility presents an ongoing hurdle in the advancement and implementation of this very promising technology.

## Data Availability

No new data were created or analyzed in this study. Data sharing is not applicable to this article.
